# Gender differences in plasma S100B levels of patients with major depressive disorder

**DOI:** 10.1186/s12888-024-05852-7

**Published:** 2024-05-23

**Authors:** Yifan Wu, Yihui Lu, Lingtao Kong, Yu Xie, Wen Liu, Anqi Yang, Kaiqi Xin, Xintong Yan, Longhai Wu, Yilin Liu, Qianying Zhu, Yang Cao, Yifang Zhou, Xiaowei Jiang, Yanqing Tang, Feng Wu

**Affiliations:** 1 Department of Psychiatry, The First Hospital of China Medical University, 155 Nanjing North Street, 110001 Liaoning, P.R. China; 2 Faculty of Public Health, China Medical University, 110001 Liaoning, P.R. China; 3 Shenyang Mental Health Center, 110001 Liaoning, P.R. China; 4 Brain Function Research Section, Department of Radiology, The First Hospital of China Medical University, 110001 Liaoning, P.R. China; 5 Department of Geriatric Medicine, The First Hospital of China Medical University, 110001 Liaoning, P.R. China

**Keywords:** Plasma S100B, Gender difference, Major depressive disorder, Mann–Whitney test, Scheirer-Ray-Hare test, Glial cells

## Abstract

**Background:**

Low concentrations of S100B have neurotrophic effects and can promote nerve growth and repair, which plays an essential role in the pathophysiological and histopathological alterations of major depressive disorder (MDD) during disease development. Studies have shown that plasma S100B levels are altered in patients with MDD. In this study, we investigated whether the plasma S100B levels in MDD differ between genders.

**Methods:**

We studied 235 healthy controls (HCs) (90 males and 145 females) and 185 MDD patients (65 males and 120 females). Plasma S100B levels were detected via multifactor assay. The Mahalanobis distance method was used to detect the outliers of plasma S100B levels in the HC and MDD groups. The Kolmogorov–Smirnov test was used to test the normality of six groups of S100B samples. The Mann–Whitney test and Scheirer-Ray-Hare test were used for the comparison of S100B between diagnoses and genders, and the presence of a relationship between plasma S100B levels and demographic details or clinical traits was assessed using Spearman correlation analysis.

**Results:**

All individuals in the HC group had plasma S100B levels that were significantly greater than those in the MDD group. In the MDD group, males presented significantly higher plasma S100B levels than females. In the male group, the plasma S100B levels in the HC group were significantly higher than those in the MDD group, while in the female group, no significant difference was found between the HC and MDD groups. In the male MDD subgroup, there was a positive correlation between plasma S100B levels and years of education. In the female MDD subgroup, there were negative correlations between plasma S100B levels and age and suicidal ideation.

**Conclusions:**

In summary, plasma S100B levels vary with gender and are decreased in MDD patients, which may be related to pathological alterations in glial cells.

## Introduction

Major depressive disorder (MDD) is a widespread and disabling major psychiatric disorder [1, 2], characterised by clinical manifestations such as persistent depressed mood, diminished interest, reduced volitional activity, somatisation symptoms, weight changes, cognitive dysfunction, and sleep disturbances [1]. The neurological origins and pathogenic processes of MDD remain unknown as of this writing. However, a study found that alterations of neurons and glial cells are an important factor in the histopathological mechanisms of MDD [3].

Over the past decade, researches have linked astrocyte pathology to the emergence of mood disorders, such as MDD [4, 5]. Studies have shown that S100B is an important factor influencing the pathophysiological and histopathological processes of MDD [6–10]. The S100 protein family, containing S100B, a 21 kDa calcium-binding protein, was first isolated from bovine brain by Moore in 1965 and named for its 100% solubility in ammonium sulphate [11]. It exists in vivo in different sub-chain forms, including the α and β forms, with the β form (96%) predominantly found in the brain [12]. In mammals, S100B is most abundant in glial cells of the central and peripheral nervous system [13–15] and is especially expressed in glial cells in the brain [16]. The production and release of S100B in glial cells are triggered mainly by extracellular 5-hydroxytryptamine (5-HT) binding to 5-HT1A receptors on astrocytes [17, 18], serving as a peripheral biochemical marker of neural injury with potential effects including reactive gliosis, astrocyte death, and blood–brain barrier (BBB) dysfunction [19]. Extracellularly, S100B is a bimodal neurotrophic factor. Low concentrations (nanomolar) of S100B promote axonal extension and protect neuronal survival [20], while higher concentrations (micromolar) induce apoptosis of neurons and astrocytes [21]. Huttunen et al. [22] suggested that the receptor of advanced glycation end products (RAGE) is a signalling receptor for the trophic and toxic effects of S100B. The primary roles of S100B in morphogenesis include glial cell proliferation and maturation, axon extension, and synaptic development [18, 19, 23], which may involve mitogen-activated protein kinase (MAPK) [24] and activation of RAGE in neurons [22]. Thus, S100B may be involved in coordinating the development and maintenance of the central nervous system (CNS), like other glia-derived cytokines, by synchronising the stimulation of neuronal differentiation and astrocyte proliferation. The expression of S100B and RAGE can be reduced by stress and depression, and these effects can be reversed or prevented by antidepressant treatment [7]. In several brain regions, including the hippocampus, a decrease in the number of S100B-positive cells and a decrease in glial fibrillary acidic protein (GFAP) expression indicate a significant reduction in glial cell density [25]. Animal studies have shown reduced S100B levels in cerebrospinal fluid (CSF), the hippocampus [26], and the prefrontal cortex [27] of rats after chronic, unpredictable stress and in the hippocampus of pregnant rats after exposure to stress [28].

Studies have shown gender differences in depression [29], which may be related to an earlier age of onset, higher anxiety levels, and lower alcohol use in women [30]. It has also been demonstrated that there are gender differences in serum neurotrophic factors in adolescents with depression and that they correlate with clinical severity [31]. Our recent study found that there was a gender difference in plasma glial cell line-derived neurotrophic factor levels of patients within bipolar disorder [32]. A fundamental finding arising from recent studies on the physiological role of microglia in neural development is their sexual dimorphism, suggesting that there is gender dimorphism in the underlying mechanisms of MDD [33]. A large-scale gene expression meta-analysis found that male MDD patients exhibit increased genes associated with oligodendrocytes and microglia, while markers for these cell types are reduced in female MDD patients [34]. Females from 13 to 15 years are prone to depression when they reach puberty too early, which may be related to hormone levels [35]. Chronic stress causes astrocyte atrophy in males and astrocyte hypertrophy in females, and gonadal hormones modulate this difference to some extent [36]. Evidence suggests that certain brain structures and functions are different at different ages [37, 38]. A survey analysing age and depression found that the morbidity of depression began to decline in early adulthood and reached the lowest level in middle age, around age of 45 years [39]. A previous study found that serum S100B levels were significantly higher in females with MDD than in males [8], but it had a small sample size and too many confounding variables, and it is unclear whether gender differences in plasma S100B levels also exist in patients with MDD.

Considering the above reasons, including hormones, age, and brain maturation, in this study, we investigated whether plasma S100B expression in MDD patients and healthy controls (HCs) under the age of 45 differed significantly by gender. We hypothesised that S100B levels may be differentially altered in male and female patients. The results of this study may provide a reference for studying the pathophysiological mechanisms of MDD across gender.

## Methods and materials

### Participants

Socio-demographics assessment: We used uniform criteria for inclusion of participants information, including but not limited to: gender, age, height, weight, education, ethnicity, handedness, and so on.

#### Patient group (MDD group)

Data for all patients in this study were collected at the psychiatric outpatient clinic of the First Hospital of China Medical University from 2011 to 2022. They were all diagnosed with MDD after completing a structured clinical interview for the Diagnostic and Statistical Manual of Mental Disorders (DSM-IV-TR) (SCID) [40] by two experienced psychiatrists. All of the participants fit the DSM-IV-TR diagnostic criteria for MDD and had no history of additional DSM-IV-TR axis I disorders (including schizophrenia, bipolar disorder, or substance abuse). Those with head trauma, brain haemorrhage, brain infarction, dementia, and other organic diseases (e.g., hypertension, diabetes, immune system disorders, and infectious diseases within one month) were excluded.

#### Clinical variables

Disease duration: Total time from the patient's first diagnosis of MDD by SCID to the day of enrolment; Assessment and grouping of suicidal ideation: The Beck questionnaire for suicidal ideation (Beck scale for suicide ideation, BSI) was used [41]. The scale was selected in this study to assess the patients' severity of suicidal ideation. It consists of 19 items, and the higher the score, the stronger the suicidal ideation, and the higher the risk of suicide. If the answer to items 4 (active suicidal ideation) and 5 (passive suicidal ideation) is "none", the patient is considered to have no suicidal ideation and is classified as having no suicidal ideation, otherwise he/she is classified as having suicidal ideation; Assessment and grouping of suicidal behaviour: If the patient had made at least one suicide attempt at any time in his/her life, he/she is classified as having suicidal behaviour, otherwise he/she is classified as having no suicidal behaviour; First-onset: Patients’ first depressive episode can meet the DSM-IV-TR criteria for MDD. No other DSM-IV-TR Axis I diagnosis; Medications: Of the 185 participants with MDD, 55 (29.7%) were medication-naïve. The remaining 130 participants were prescribed psychiatric medications, including antidepressants (170, 91.9%), anxiolytics (102, 55.1%), mood stabilisers (29, 15.7%), atypical antipsychotics (17, 9.2%), and traditional Chinese medicine (50, 27.0%). Fifteen (8.1%) of the medication users had unknown specific medications.

#### Healthy control group (HC group)

A total of 235 participants were enrolled through voluntary social recruitment. Individuals with DSM-IV-TR axis disorders or a history of cranial trauma, cerebral haemorrhage, cerebral infarction, dementia, or other organic diseases (including hypertension, diabetes, immune system disorders, and infectious diseases within one month) were excluded.

The MDD group consisted of 185 patients, comprising 65 males (male MDD, mMDD) and 120 females (female MDD, fMDD), while the HC group consisted of 235 participants, with 90 male participants (male HC, mHC) and 145 female participants (female HC, fHC). All 420 participants’ clinical symptoms were assessed with the 17-item Hamilton Depression Rating Scale (HAMD-17)  [42] and Hamilton Anxiety Rating Scale (HAMA) [43] before collecting blood samples. All study participants signed an informed consent form that was consistent with the trial content and procedures. The China Medical University's Ethics Committee had given the study their approval.

### Measure of S100B

We collected participants’ blood samples in vacuum tubes and centrifuged the samples at 2 000 rpm/min for 10 min. The separated plasma samples were stored uniformly in a refrigerator at − 80 °C for subsequent testing. Luminex multifactor assay technology was used to measure the S100B concentration in the plasma samples in strict accordance with the kit instructions (R&D Systems, Minneapolis, Minnesota, USA). All samples were assayed twice, using the same assay equipment and by the same laboratory personnel.

### Data analysis

The Mahalanobis distance method was used to detect the outliers of plasma S100B levels in the HC and MDD groups. The Kolmogorov–Smirnov test was used to test the normality of six groups of S100B samples, comprising HC, MDD, and the four subgroups (mHC, fHC, mMDD, and fMDD), and found that all six groups had abnormal distribution (*p* < 0.05). Therefore, the Mann–Whitney test and Scheirer-Ray-Hare test were used for the comparison of plasma S100B levels between diagnoses and genders. Furthermore, the Spearman correlation analysis was done to evaluate whether there was a correlation between plasma S100B and demographic information such as age and education year as well as clinical characteristics such as disease duration, medication use, and HAMD-17 scores. *P* < 0.05 was considered statistically significant.

Since the plasma S100B levels did not follow a normal distribution, we used two-way non-parametric analysis of variance (ANOVA), which was based on the basic idea and principle of the Scheirer-Ray-Hare test [44–46], and compiled the rank of the original data. If the H_0_ hypothesis was established, i.e., if there was no main effect of group (factor A) and gender (factor B) or interaction effect between A and B, the rank of the observations was a random sample whose random error satisfied an independent distribution whose mean was 0 and variance was *σ*^2^_e_ [*σ*^2^_e_ = *n* (*n* + 1)/12, where *n* is the total sample size]. According to the idea of ANOVA, we used rank as the outcome variable. The total variance of the rank was decomposed into the variance of factor A (RSS_A_), the variance of factor B (RSS_B_), and the variance of the interaction between A and B (RSS_AB_), and the degrees of freedom (ν) were also decomposed. Then we constructed the test statistic H, which approximately followed the *χ*^2^ distribution with certain degrees of freedom. If H_0_ was established, the value of H was not large, and by checking the *χ*^2^ boundary value table, the probability *p* corresponding to the value of H could be determined to realise the inference of the interaction effect of A-factor, B-factor, and AB-factor. H_A_ = RSS_A_/*σ*^2^_e_, ν_A_ = A-factor levels − 1; H_B_ = RSS_B_/*σ*^2^_e_, ν_B_ = B-factor levels − 1; and H_AB_ = RSS_AB_/*σ*^2^_e_, ν_AB_ = (A-factor levels − 1) × (B-factor levels − 1). The following analyses were performed in IBM SPSS Statistics for Windows, version 22.0 (IBMCorp., Armonk, N.Y., USA):

1. Data were entered into SPSS.

2. Mixed rank coding was performed.

3. Two-way ANOVA was performed on the rank (variable RS100B) to obtain the diagnostic factor (group), gender factor (gender), interaction effect (group × gender), and SS_corrected-total_ and degrees of freedom of the corrected total variance.

4. The corrected total mean square (S^2^_corrected-total_) was calculated: S^2^_corrected-total_ = SS_corrected-total_/ν_corrected-total_, S^2^_corrected-total_ ≈ *σ*_e_^2^ = *n* (*n* + 1)/12.

5. The H statistic was calculated: H_group_ = SS_group_/S^2^_corrected-total_; H_gender_ = SS_gender_/S^2^_corrected-total_; H_group × gender_ = SS_group × gender_/S^2^_corrected-total_.

6. The probability *p* of the H statistic was determined. The H statistic obeyed the *χ*^2^ distribution [44, 45], so the probabilities could be determined using the CDF CHISQ(*χ*^2^, *ν*) function provided by SPSS (Table 2).

## Results

### Comparison of the demographics and clinical characteristics

The HC and MDD groups were not statistically different in terms of age (*t* = 1.55, *p* = 0.122), gender (*χ*^2^ = 0.45, *p* = 0.505), Body Mass Index (BMI) (*t* = –0.33, *p* = 0.741), education (*t* = 1.67, *p* = 0.095), ethnicity (*χ*^2^ = 0.21, *p* = 0.644), or handedness (*χ*^2^ = 1.10, *p* = 0.293). Among the four subgroups, we did not find a statistical difference between mMDD and mHC, fMDD and fHC, mMDD and fMDD, or mHC and fHC (*p* > 0.05) in terms of age and education, but in terms of BMI, there were significant difference between mHC and fHC (*t* = 4.46, *p* < 0.001), mMDD and fMDD (*t* = 3.41, *p* < 0.001). Between the mMDD and fMDD subgroups, there was no significant difference in terms of duration (*t* = 0.81, *p* = 0.418), first-onset (*χ*^2^ = 0.61, *p* = 0.434), and medication-naïve (*χ*^2^ = 0.61, *p* = 0.434). The HAMD-17 scores and HAMA scores differed significantly between the HC and MDD groups (*t* =  − 33.12, *p* < 0.001) and the other two subgroups (mHC and mMDD [*t* =  − 33.12, *p* < 0.001], fHC and fMDD [*t* =  − 27.82, *p* < 0.001]), but they were not significantly different between mHC and fHC or mMDD and fMDD (*p* > 0.05) (Table 1).

**Table 1:** Demographic and clinical characteristics of HC and MDD participants

	HC (*n* = 235)	MDD (*n* = 185)			*t*/χ^2^/Z			*p*
	Male	Female	Male	Female				
**Demographic**								
Gender	90	145	65	120		0.45		> 0.05
Age (years)	26.58 ± 6.33	26.10 ± 6.77	24.71 ± 6.71	25.49 ± 7.60		0.54^a^, -0.70^b^		> 0.05^a, b^
						1.77^c^, 0.68^d^		> 0.05^c, d^
Education (years)	14.21 ± 3.47	14.62 ± 2.60	13.78 ± 2.53	14.13 ± 2.62		-0.96^a^, -0.88^b^		> 0.05^a, b^
						0.89^c^, 1.51^d^		> 0.05^c, d^
BMI(kg/m^2^)	23.54 ± 4.21	21.21 ± 3.36	23.79 ± 4.85	21.40 ± 4.38		4.46^a^, 3.41^b^		< 0.001^a, b^
						-0.34^c^^,^ -0.41^d^		> 0.05^c, d^
Ethnic, Han	208/27		161/24			0.21		> 0.05
Handedness, Right	222/13		170/15			1.10		> 0.05
**Clinical**								
Duration (months)	**—**		23.54 ± 26.19	20.03 ± 29.12		0.81^b^		> 0.05^b^
First-onset, yes	**—**		48/17	82/38		0.61^b^		> 0.05^b^
Medication-naïve, yes	**—**		17/48	38/82		0.61^b^		> 0.05^b^
Medication								
Antidepressants	**—**		61	109		**—**		**—**
Anxiolytics	**—**		36	76		**—**		**—**
Antipsychotics	**—**		4	13		**—**		**—**
Mood stabilizer	**—**		6	23		**—**		**—**
Chinese medicine	**—**		7	43		**—**		**—**
Unknown	**—**		4	11		**—**		**—**
HAMD-17	1.08 ± 1.50	1.25 ± 1.63	18.85 ± 6.87	20.16 ± 7.77		-0.80^a^, -1.14^b^		> 0.05^a, b^
						-20.50^c^, -17.60^d^		< 0.0001^c, d^
HAMA	1.20 ± 1.70	1.04 ± 1.53	19.62 ± 8.33	18.79 ± 8.89		0.74^a^, 0.64^b^		> 0.05^a, b^
						-26.20^c^, -21.61^d^		< 0.0001^c, d^
Suicidal ideation	**—**		32	73		**—**		**—**
Suicidal behavior	**—**		15	38		**—**		**—**
S100B (pg/ml)								
Median	153.82	116.63	116.98	80.63			-1.84^a^, -2.78^b^	> 0.05^a^
Interquartile range	77.49 ~ 321.97	58.78 ~ 244.03	61.02 ~ 187.28	32.64 ~ 134.30			-2.39^c^, -4.32^d^	< 0.01^b^ < 0.05^c^ < 0.0001^d^

^a^Male HC compared with Female HC;

^b^Male MDD compared with Female MDD;

^c^Male HC compared with Male MDD;

^d^Female HC compared with Female MDD

*MDD* major depressive disorder, *HC* health controls, *n*=number, Values are expressed as Mean ± SD, median and interquartile range, *BMI* body mass index, *HAMD-17* 17-item Hamilton Depression Rating Scale, *HAMA* Hamilton Anxiety Rating Scale

The Mann–Whitney test was used to compare plasma S100B levels between different groups. The S100B levels in the MDD group were significantly lower than those in the HC group (*Z* =  − 4.85, *p* < 0.0001). Within the MDD group, the S100B levels showed no significant difference between the first-onset group and the recurrent group (*Z* =  − 1.39, *p* = 0.166). The S100B levels in the medication-naïve group were not significantly different from those in the medication group (Z =  − 0.94, *p* = 0.349). In terms of subgroups, the S100B levels in the mMDD were significantly higher than those in the fMDD (*Z* =  − 2.78, *p* = 0.005). The S100B levels in the mHC were significantly higher than those in the mMDD (*Z* =  − 2.39, *p* = 0.017). The S100B levels in the fHC were significantly higher than those in the fMDD (*Z* =  − 4.32, *p* < 0.0001), whereas the S100B levels in the mHC were not significantly different from those in the fHC (*Z* =  − 1.84, *p* = 0.066) (Table 1).

### Results of the Scheirer-Ray-Hare test for comparison of S100B

The two-way non-parametric ANOVA revealed that the interaction of gender and group did not have a significant effect on plasma S100B levels (H = 0.67, *p* = 0.437, *η*^2^ = 0.002). The S100B levels were significantly lower in the MDD group than in the HC group (H = 18.93, *p* = 0.021, *η*^2^ = 0.047), and the difference between genders was also significant, with significantly higher S100B levels in males than in females with MDD (H = 10.06, *p* < 0.001, *η*^2^ = 0.025) (Table 2).

**Table 2:** The results of Scheirer-Ray-Hare test for Comparison of plasma S100B levels

Source of Variance	Sum of Squares	df	Mean Square	F	H	*p*	*η*^*2*^
Group	278936.58	1	278936.58	20.44	18.93	0.0210	0.047
Gender	148187.44	1	148187.44	10.86	10.06	0.0009	0.025
Group × Gender	9844.96	1	9844.96	0.72	0.67	0.4367	0.002
Error	5675967.74	416	13644.15				
Total	24783411.00	420					
Corrected Total	6173106.00	419					

### Spearman correlation of plasma S100B levels with the demographics and clinical characteristics in the MDD group

In the MDD group, we did not find any significant difference between plasma S100B levels and age (*r* = –0.09, *p* = 0.208), education (*r* = 0.01, *p* = 0.875), BMI (*r* = 0.02, *p* = 0.748), duration (*r* = 0.04, *p* = 0.630), first-onset (*r* = 0.102, *p* = 0.167), medication-naïve (*r* = –0.069, *p* = 0.350), suicidal ideation (*r* = 0.09, *p* = 0.225), suicidal behaviour (*r* = 0.12, *p* = 0.114), HAMD-17 scores (*r* = 0.06, *p* = 0.394), or HAMA scores (*r* = 0.06, *p* = 0.458).

**Fig. 1:**
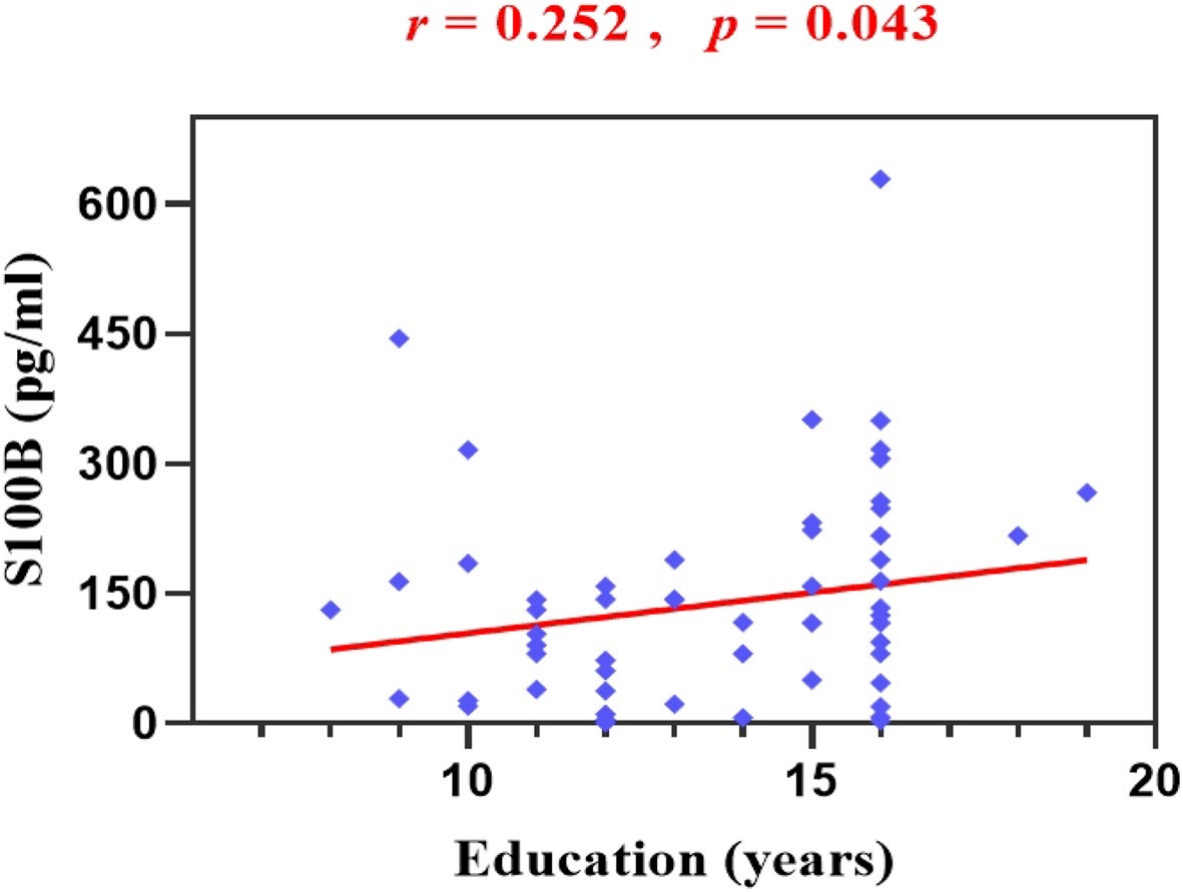
The mMDD group showed a positive correlation between education and plasma S100B levels (*r* = 0.252, *p* = 0.043). *p < 0.05 was considered statistically significant

In the mMDD subgroup, there was a positive correlation between plasma S100B levels and education (*r* = 0.25, *p* = 0.043). There was no significant correlation of S100B levels with age (*r* = 0.18, *p* = 0.141), BMI (*r* = –0.06, *p* = 0.663), duration (*r* = 0.20, *p* = 0.120), first-onset (*r* = 0.18, *p* = 0.164), medication-naïve (*r* = 0.08, *p* = 0.530), suicidal ideation (*r* = 0.02, *p* = 0.871), suicidal behaviour (*r* = 0.17, *p* = 0.170), HAMD-17 scores (*r* = 0.21, *p* = 0.094), or HAMA scores (*r* = 0.09, *p* = 0.133) (Table 3; Table 4; Fig. 1).

**Table 3:** Correlation of S100B levels with demographic characteristics

	BMI	Age			Education years	
	*r*	*p*	*r*	*p*	*r*	*p*
MDD	0.024	0.748	-0.093	0.208	0.012	0.875
mMDD	-0.055	0.663	0.184	0.141	0.252	0.043*
fMDD	-0.031	0.735	-0.204	0.025*	-0.117	0.203

*MDD* major depressive disorder, *mMDD* male major depressive disorder, *fMDD* female major depressive disorder, *BMI* body mass index. *p < *0.05 was considered statistically significant*

**Table 4:** Correlation of S100B levels with clinical characteristics

	HAMD-17		HAMA		Suicidal ideation	
	*r*	*p*	*r*	*p*	*r*	*p*
MDD	0.063	0.394	0.055	0.458	0.090	0.225
mMDD	0.209	0.094	0.188	0.33	0.021	0.871
fMDD	0.008	0.933	-0.029	0.752	-0.187	0.041*

*MDD* major depressive disorder, *mMDD* male major depressive disorder, *fMDD* female major depressive disorder, *HAMD-17* 17-item Hamilton Depression Rating Scale, *HAMA* Hamilton anxiety rating scale. *p < 0.05 was considered statistically significant

**Fig. 2:**
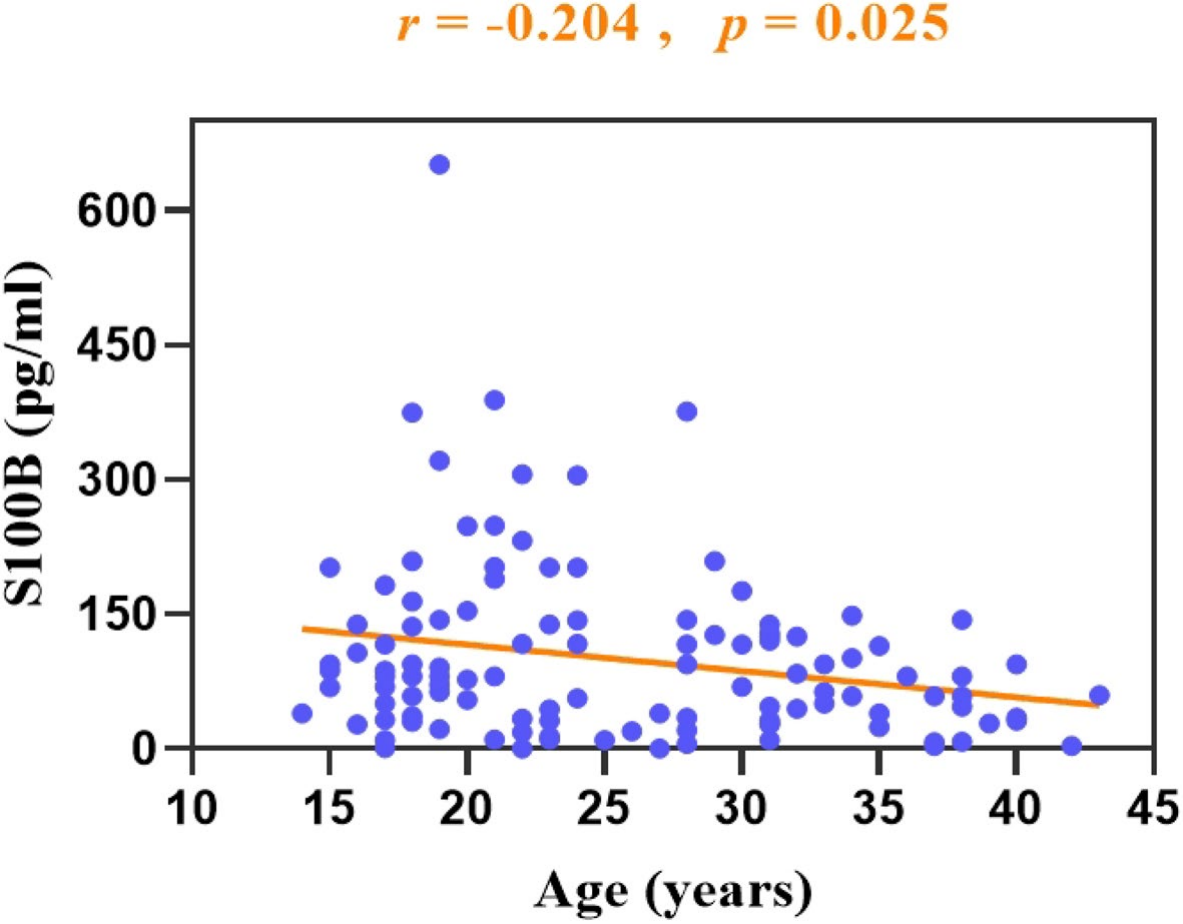
The fMDD group showed a negative correlation between age and plasma S100B levels (*r* = -0.204, *p* = 0.025). *p < 0.05 was considered statistically significant

In the fMDD subgroup, there was a negative correlation between plasma S100B levels and age (*r* = –0.20, *p* = 0.025). There was a positive correlation between S100B levels and suicidal ideation (*r* = 0.19, *p* = 0.041). There was no significant correlation of S100B levels with education (*r* = –0.12, *p* = 0.203), BMI (*r* = –0.03, *p* = 0.735), duration (*r* = –0.10, *p* = 0.288), first-onset (*r* = 0.04, *p* = 0.699), medication-naïve (*r* = –0.14, *p* = 0.127), suicidal behaviour (*r* = 0.12, *p* = 0.177), HAMD-17 scores (*r* = 0.01, *p* = 0.933), or HAMA scores (*r* = –0.03, *p* = 0.752) (Table 3; Table 4; Fig. 2).

## Discussion

There was a significant difference in plasma S100B levels between the MDD and HC groups in our study. Among subgroups, S100B levels were significantly lower in females than in males in the MDD group, while in the HC group, there was no significant difference in S100B levels between genders. In both the male and female groups, S100B levels were significantly lower in MDD than in HC. Therefore, it could be inferred that the decrease in plasma S100B levels in the MDD group is greater in females compared to males. These findings suggest the importance of gender for plasma S100B expression in patients with MDD.

Astrocyte is the major type of glial cells that plays a crucial role in the CNS and can be identified by the expression of specific markers, such as glial fibrillary acidic protein (GFAP) and S100B, as well as by their morphology [47, 48]. Previous studies have linked astrocyte pathology to the development of mood disorders [4, 5]. Histopathological analysis of autopsy brain samples from MDD patients revealed a considerable reduction in glial cell density, as evidenced by a decrease in the number of cells expressing S100B [49], as well as a momentous reduction in GFAP expression in several brain regions, including the hippocampus [50]. The reduction in S100B protein expression levels in the hippocampal region due to chronic stress exposure can be turned back with treatment by fluoxetine [51]. Studies have shown that the reduction in astrocyte number and morphology terribly impairs neurophysiology and function, especially in cognitive tasks [52]. This explains why the majority of MDD patients have reduced cognitive function. Previous reports shown that chronic stress could cause astrocyte shrinkage by reducing the length, branching, and volume of protrusions, suggesting that astrocytes play a role in the reduction of hippocampal volume as the duration of MDD increases [53–55]. Preclinical studies using animal models of depression have consistently shown a decrease in the number and expression of cells expressing S100B and GFAP [53, 56–61], including in the hippocampal dentate gyrus (DG) [62, 63]. This explains why plasma S100B was significantly reduced in MDD patients in our study.

S100B, as a critical neurotrophic factor for serotonergic neurons, can regulate sprouting and synaptic plasticity in the adult brain [64, 65]. After 5-HT binding activates 5-HT1A receptors on the surface of astrocytes, astrocytes release the neurotrophic factor S100B [17, 64, 65]. Thus, 5-hydroxytryptaminergic neurons can regulate the sprouting and regeneration of their nerve endings by triggering the release of S100B [17]. S100B may balance the amount of 5-HT released into the synaptic gap and rapidly inactivate it, so that high levels of S100B may correspond to a 5-HT ‘low synaptic state’, representing a reduction in its ability to precisely regulate specific neuronal circuits [66]. Therefore, we speculate that the decrease in S100B levels in MDD patients may be a compensatory mechanism to repair or reverse the impairment in neurons and glial cells caused by the disease. A follow-up study from the Netherlands [67] found that the activation of the inflammatory response system and patterns of change in chemokines and S100B, are essential in the events leading to the development of mood disorders. It may be related to tissue migration and hyperactivity of monocytes.

Gender differences in S100B concentrations in cord blood were first observed by Gazzolo et al. [68] and were further confirmed by the results of another of their studies of blood S100B in children [69]. These differences can be explained by the different brain maturation patterns of the two sexes [70, 71]. In contrast, in another study of late preterm infants, S100B protein concentrations in the urine of female infants were found to be higher than in males at all weeks of gestation [72]. Another study found that the reference level of capillary S100B in females aged 1 to 2 years was more than twice that of males in the same age group [73]. Previous studies have shown that serum S100B levels are significantly higher in females than in males in the schizophrenic population [74]. A prospective study of patients with COVID-19 showed that plasma brain injury markers, including S100B, were higher in males than in females, but this difference was not statistically significant [75]. It has been shown that, in addition to astrocytes, adipocytes also release S100B. Furthermore, insulin has been reported to reduce S100B levels in both adipocytes and astrocytes [76]. Interestingly, overweight, visceral obesity, and insulin resistance may be associated with S100B levels in patients with schizophrenia [77]. In our study, although not statistically significant, females possessed a lower BMI than males, previous studies have shown that neuroinflammation may be a modifiable pathway linking obesity and depression [78], and obesity may trigger persistent epigenetic changes in innate immunity and exacerbates neuroinflammation, which may explain why our findings showed lower S100B in females [79]. Thus, S100B may be associated with altered adipocyte function in addition to being a marker of abnormal astrocyte function.

Previous studies have found that S100B concentrations in the urine of late preterm infants correlate with gestational age [72]. In foetal cord blood, there is a significant correlation between S100B and gestational age [68]. In the peripheral blood of children, there is also a correlation between S100B and age [69], which is consistent with our findings in the fMDD subgroup. Although some studies have found no significant difference in serum S100B levels between patients with and without a history of suicidal mood disorders, different results were obtained by Dogan et al. [80], who found higher CSF S100B levels in the suicidal group than in the non-suicidal group. Our study found that plasma S100B was positively associated with suicidal ideation in patients with MDD. This is consistent with the findings of Falcone et al. [81], who found that serum S100B is a marker of suicidal ideation in adolescents. Around the world, females are at a higher risk of attempting suicide [82, 83], which seems to validate our results indicating that the correlation between S100B and suicidal ideation is more pronounced in female MDD patients.

### Limitations

There are certain limitations in our study. First, it was a cross-sectional study, and longitudinal studies with follow-up of patients may be more likely to show changes in plasma S100B levels. Second, the number of study subjects varied across groups. In particular, the number of patients in the MDD group was lower than that in the HC group, and there were fewer male MDD patients than females. In addition, although our results showed no significant difference in S100B levels between the medication and medication-naïve groups, a previous study found that psychotropic medications (e.g., mood stabilisers, antidepressants, and antipsychotics) may affect the expression and/or secretion of S100B [84]; we were unable to determine whether medications affected plasma S100B levels in our study.

## Conclusion

The results obtained in this study suggest that plasma S100B levels are significantly decreased in MDD patients and that its expression is influenced by gender, which may be related to pathological alterations in astrocytes, different pathogeneses of MDD in different genders, and different brain maturation patterns in different genders. The pathophysiological processes of the gender differences in plasma S100B among MDD patients and its effects on clinical symptoms need to be further.

investigated.

## Data Availability

The datasets used and/or analysed during the current study are available from the corresponding author on reasonable request.
